# Is the reporting timeliness gap for avian flu and H1N1 outbreaks in global health surveillance systems associated with country transparency?

**DOI:** 10.1186/1744-8603-9-14

**Published:** 2013-03-25

**Authors:** Feng-Jen Tsai, Eva Tseng, Chang-Chuan Chan, Hiko Tamashiro, Sandrine Motamed, André C Rougemont

**Affiliations:** 1 Master program in Global Health and Development, College of Public Health and Nutrition, Taipei Medical University, 250 Wu-Hsing Street, Taipei City 110, Taiwan; 2 Department of Internal Medicine, Thomas Jefferson University Hospital, Philadelphia, USA; 3 Institute of Occupational Medicine and Industrial Hygiene, Taipei, Taiwan; 4 Global Health Center, College of Public Health, National Taiwan University, Taipei, Taiwan; 5 Department of Global Health and Epidemiology, Hokkaido University Graduate School of Medicine, Sapporo, Japan; 6 Institute of Social and Preventive Medicine, University of Geneva, Geneva, Switzerland

## Abstract

**Background:**

This study aims to evaluate the length of time elapsed between reports of the same incidents related to avian flu and H1N1 outbreaks published by the WHO and ProMED-mail, the two major global health surveillance systems, before and after the amendment of the International Health Regulations in 2005 (IHR 2005) and to explore the association between country transparency and this timeliness gap.

**Methods:**

We recorded the initial release dates of each report related to avian flu or H1N1 listed on the WHO Disease Outbreak News site and the matching outbreak report from ProMED-mail, a non-governmental program for monitoring emerging diseases, from 2003 to the end of June 2009. The timeliness gap was calculated as the difference in days between the report release dates of the matching outbreaks in the WHO and ProMED-mail systems. Civil liberties scores were collected as indicators of the transparency of each country. The Human Development Index and data indicating the density of physicians and nurses were collected to reflect countries’ development and health workforce statuses. Then, logistic regression was performed to determine the correlation between the timeliness gap and civil liberties, human development, and health workforce status, controlling for year.

**Results:**

The reporting timeliness gap for avian flu and H1N1 outbreaks significantly decreased after 2003. On average, reports were posted 4.09 (SD = 7.99) days earlier by ProMED-mail than by the WHO. Countries with partly free (OR = 5.77) and free civil liberties scores (OR = 10.57) had significantly higher likelihoods of longer timeliness gaps than non-free countries. Similarly, countries with very high human development status had significantly higher likelihoods of longer timeliness gaps than countries with middle or low human development status (OR = 5.30). However, no association between the timeliness gap and health workforce density was found.

**Conclusion:**

The study found that the adoption of IHR 2005, which contributed to countries’ awareness of the importance of timely reporting, had a significant impact in improving the reporting timeliness gap. In addition, the greater the civil liberties in a country (e.g., importance of freedom of the media), the longer the timeliness gap.

## Background

The outbreak of Severe Acute Respiratory Syndrome (SARS) in 2003 alerted the world to the importance of a timely global surveillance system for public health emergencies with potential international impacts [1]. The global pandemic was exacerbated by the initial delay in information release by the Chinese government due to the fear of political and economic consequences and ended with a total of 8,422 cases and 916 deaths worldwide [2]. The world first learned of SARS via ProMED-mail, a non-governmental global electronic reporting system, on February 10, 2003, a day earlier than the official report by the World Health Organisation (WHO). However, the reports by both systems were published 3 months after the outbreak first occurred.

Previous studies have emphasised that timeliness is the key to the success of surveillance systems and to reflecting the time delay between response steps in the surveillance process [3-5]. Surveillance and reporting were the first two steps for further response in infectious disease control. However, the reporting process was found to be frequently subjected to political influence that affected the timeliness of the reporting and thus the effective initiation of public health interventions [6-9].

As the main surveillance network of the WHO, the Global Outbreak Alert & Response Network (GOARN) was established in 2000 [10]. However, its efficiency is influenced by geographical and timeliness gaps due to political concerns [11,12]. As an alternative, ProMED-mail, the non-governmental Program for Monitoring Emerging Diseases, was established in 1994 to provide early warnings about outbreaks based on information from various sources, including individuals and media reports; furthermore, unlike the WHO program, ProMED-mail does not require official clearance prior to posting reports [13]. As one of the pioneers of internet-based reporting systems, ProMED-mail is also one of the largest publicly available reporting networks [6]. Previous studies have confirmed its credibility [14-18]. In addition, the effects of reporting in a timely manner (without political constraints) and the efficiency of ProMED-mail in decreasing the delay in reporting have been studied through comparisons to the timeliness of reporting from the WHO [14-18]. Although internet-based sources of information not only allow the timely detection of outbreaks but also increase reporting transparency, these sources still cannot overcome the problems of non-transparency by authorities who deliberately conceal information.

The lack of transparency for political reasons, lack of consensus in policy and strategy and inadequate training and resources for health system personnel have all been cited as barriers to effective and timely global health surveillance [7,19]. In response to the outbreak of SARS, the 58^th^ World Health Assembly adopted a new set of International Health Regulations (IHR 2005) on May 23, 2005 to close the gaps in the global health surveillance system. As the only regulations for the global surveillance of high-priority infectious diseases, the revision of IHR 2005 responded to the needs of an effective global health surveillance system [6,20]. IHR 2005 not only included national obligations to achieve a set of core surveillance and response capacities by eliminating technical, resource, governance, legal and political obstacles in the health system, it also required members to assess any public health emergency of international concern within 48 hours and to notify the WHO within 24 hours to ensure the timely receipt of the information. In addition, the WHO was allowed to consider reports from unofficial sources, in accordance with Article 9 [21]. Because public health events overlap with trade and security issues, global health surveillance issues have attracted increasing attention. However, the effects of IHR 2005 on the reporting timeliness gap and the association between transparency and the reporting timeliness gap have yet to be evaluated.

The most pressing rationale for transparency in infectious disease reporting is that open communication and information can prevent delayed reports and responses to outbreaks [8,9]. Transparency can be represented by the extent of civil liberties and the circulation of public information. Civil liberties are the rights and freedoms that protect individuals from unfair infringement by the government of the nation in which they reside and are the basic tenets of democracy. Moreover, civil liberties set limits on the government so that its members cannot abuse their power and interfere unduly with the affairs of private citizens. Countries with strong civil liberties typically also have well developed mass media that is capable of reporting infectious disease news promptly after its occurrence [22]. Therefore, the public can receive information about a disease outbreak earlier through media or other channels in countries with better transparency. Consequently, in countries with good civil liberties, ProMED-mail might receive reports of new emerging infectious diseases from individuals or the media and release the information earlier than the WHO, which would likely receive official information from the government at a later time and post reports after receiving official clearance.

Given that the timeliness gap between WHO and ProMED-mail in a particular country might reflect the information delay due to political constraints, we conducted this study under the hypothesis that better transparency would be associated with a longer reporting timeliness gap between the official and non-official global surveillance systems. Because the avian flu and H1N1 outbreaks were the emerging global pandemics following SARS, we used the reporting of avian flu and H1N1 outbreaks as the focus of our study.

## Methods

The components of the systemic rapid assessment method, which represents the capacity for timely reporting, were analysed in previous studies with the Systemic Rapid Assessment (SYSRA) Toolkit, a framework that includes: External contexts such as demographic, economic, political, legislative, epidemiologic, socio-cultural and technological factors; stewardship, which refers to organisational systems and laboratory and drug networks; financing, resource generation and allocation; and healthcare provision and information systems [23,24]. The framework provides conceptual and analytical guidelines for the evaluation of health systems and infectious disease control programs and echoes the national responsibilities required by IHR 2005 [24]. Therefore, we collected transparency data and measurements based on this framework for further analysis.

### Data source

The study was conducted from July 2007 to March 2010. To evaluate the timeliness gap between the public and private global disease outbreak surveillance systems, we conducted a comprehensive survey, obtaining the initial release dates of the reports for each avian flu and H1N1 disease outbreak listed on the WHO Disease Outbreak News from 2003 to the end of June 2009and then matching them to the corresponding outbreak report from ProMED-mail. More specifically, we first collected all avian flu and H1N1 disease outbreak reports released on the WHO Disease Outbreak News website [25]. We then looked for the same disease incident report on the ProMED-mail website based on the information indicated in the original WHO report, including disease name, country, date of onset and other details [13]. For multi-country outbreak reports from the WHO, each country report was matched separately with its corresponding report in ProMED-mail. Outbreak reports for China, Taiwan and Hong Kong were also separated for matching. In addition to the initial outbreak reports, WHO Disease Outbreak News also posts reports that are labelled as “updates”. These reports were examined for details indicating the spread of the initial disease outbreak to other regions of the affected country. Where there was new information about the outbreak spreading to other areas, we searched ProMED-mail for a matching report. Updates that mentioned only an increased number of cases without additional information about geographical spread were excluded, as were reports about WHO technical meetings and epidemiological survey findings. Additional information about the release date and source of the reports in ProMED-mail were also collected for further analysis.

Using this method, we collected a total of 423 matched reports. After excluding the reports that ProMED-mail received from the WHO, 322 reports were included in the final analysis.

The research protocol was approved by the Institutional Review Board of the College of Public Health, National Taiwan University.

### Measurements

Civil liberties scores from the Freedom House were collected as indicators of transparency for each country. The Freedom House is a renowned nongovernmental organisation that supports democracy and freedom around the world. This group evaluates the political rights and civil liberties of each country on an annual basis. In our study, we used only civil liberties as an index of transparency. The 4 key areas of information gathered for civil liberties, based on a checklist of 15 questions, include freedom of expression and belief (4 questions), associational and organisational rights (3 questions), rule of law (4 questions) and personal autonomy and individual rights (4 questions). The total number of points on the civil liberties checklists determines the civil liberties rating on a scale of 1 to 7, with 1 representing the highest degree of freedom and 7 representing the lowest. The details of the method are described in the methodology section of the Freedom House website [26]. Although we collected the historical datasets on civil liberties scores as well as the latest scores, from 2003, we used the mean of the scores from 2003 to 2007 as each country’s overall civil liberties score. We further divided the analysed countries into free, partly free and not free countries according to these scores. Countries with civil liberties scores of 1 and 2 were designated as free countries, between 3 and 5 as partly free countries, and of 6 and 7 as not free countries.

In the framework of the Systemic Rapid Assessment Toolkit, the Human Development Index (HDI) and the density of physician and nurses are used as indicators. Therefore, we collected the HDI from the United Nations Development Program (UNDP) websites and physician and nurse density data from the WHO website for further analysis [27,28]. Comprehensive data regarding IT and communication infrastructure were not available for every country, so we did not include this parameter in the study.

Human development is defined as encompassing three dimensions: population health and longevity, as measured by life expectancy at birth; knowledge and education, as measured by the adult literacy rate and the combined primary, secondary, and tertiary gross enrolment ratio; and standard of living, as measured by the natural logarithm of gross domestic product per capita (GDP) at purchasing power parity. These indicators were collected mainly from official statistics. The indexes of the three dimensions were then expressed as a value between 0 and 1 by applying a general formula. Then, the human development index was calculated as a simple average of the dimension indexes, with 1 representing the highest degree of development and 0 being the lowest. Because the human development index cannot be compared historically, we used the human development indices from 2005 to represent the human development status of each country; the human development index remained stable from 2003 to 2007. The detailed methods used to determine each value are described in the Technical Notes section of the report. In addition, the categories of very high, high, medium or low development countries used by the UN were also used in the study.

Information about the density of physician and nurses each country was further collected from the WHO website. The sum of these two numbers was calculated and used as the index for the health workforce in this study. We then categorised countries as having a high, middle, or low health workforce index according to the sum of the density of physician and nurses in each country. Countries with health workforce density scores within the upper, middle and lower tertiles were defined as high, middle and low health workforce countries, respectively.

### Data analysis

The timeliness gap between the WHO and ProMED-mail reports was calculated as the difference in days between the report release dates for the same (matched) outbreaks. The timeliness gap was further divided into two groups with a cut-off point of 3 days, is in accordance with IHR 2005, which requires countries to assess and report events to the WHO within 72 hours. Logistic regression was then adopted to estimate the association between the timeliness gap and civil liberties, human development and health workforce indices, after controlling for the year.

All statistical analysis was performed in SPSS version 18.0 software.

## Results

### Timeliness gap by year

**Figure 1:**
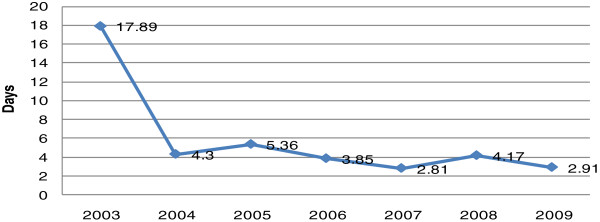
Reporting timeliness gap between WHO and ProMED-mail for avian flu and H1N1 outbreaks by year, 2003–2009.

The results for the timeliness gap by year, indicating how many days earlier events were reported by ProMED-mail than by the WHO, is shown in Figure 1. Among the 322 outbreak reports, 272 were avian flu related, and 50 were H1N1 related. The timeliness gap ranged from -37 to 54 days, i.e., reports in ProMED-mail appeared as many as 54 days earlier than the same reports from the WHO. The overall average timeliness gap of avian flu and H1N1 outbreak reports from the WHO and ProMED-mail from 2003 to July 31, 2009 was 4.09 (SD = 7.99) days. The average timeliness gap of avian flu-related outbreak reports was 4.96 days, while the average timeliness gap of H1N1 outbreak reports was 2.26 days.

### Correlations between timeliness gap and civil liberties, human development and health workforce indices

**Figure 2:**
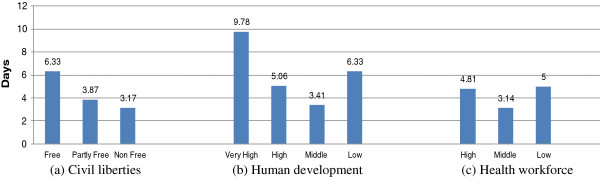
Reporting timeliness gap between WHO and ProMED-mail for avian flu and H1N1 outbreaks in countries classified by civil liberties (a), human development (b) and health workforce (c) indices.

Figure 2 shows the reporting timeliness gaps in countries as classified by civil liberties, human development status and health workforce. The study results (Figure 2a) showed that the average reporting timeliness gap of avian flu and H1N1 outbreaks was negatively associated with civil liberties rating; longer timeliness gaps were found in the more liberal countries. The longest reporting timeliness gaps for avian flu and H1N1 outbreaks were observed in free countries, followed by partially free countries, while the shortest timeliness gaps were in countries with low freedom.

The average reporting timeliness gaps for avian flu and H1N1 outbreaks showed a curved relationship with human development status (Figure 2b). The longest timeliness gaps were in countries with very high human development scores, while the shortest timeliness gaps were in countries with medium human development scores, and the second longest timeliness gaps were found in countries with low human development scores.

The average reporting timeliness gaps for avian flu and H1N1 outbreaks also showed a curved relationship with the health workforce status (Figure 2c). The timeliness gaps in countries with high and low health workforce densities were 4.81 days and 5 days, respectively, while the shortest timeliness gaps were observed in countries with medium health workforce density (3.14 days).

A total of 206 cases from 43 countries were considered to be reported in a timely fashion (timeliness gap shorter than 3 days), while 116 cases from 37 countries had a timeliness gap of longer than 3 days.

The correlations between reporting timeliness gap and civil liberties, human development and health workforce indices, as determined by logistic regression, are shown in Table 1. After controlling for the year, the reporting timeliness gap was found to be significantly associated with civil liberties and human development. Compared with non-free countries, partly free countries had a significantly higher likelihood of having a longer timeliness gap for reports of avian flu and H1N1 outbreaks (OR = 5.77), and free countries had a 10 times higher likelihood of having a longer timeliness gap than non-free countries (OR = 10.57). Similarly, countries with very high human development status had significantly higher likelihoods of longer timeliness gaps than countries with middle and low human development status (OR = 5.30). In other words, the greater the civil liberties, the longer the reporting timeliness gap between WHO and ProMED-mail for avian flu and H1N1 outbreaks.

**Table 1:** Associations between timeless gap and Civil liberties, Human development and Health workforce

**Variable^^**	**OR (95% CI)**	**p value^**
**Civil liberties**		
Non-Free		
Party Free	5.77 (1.07-31.19)	0.04^*^
Free	10.57(1.71-65.20)	0.01^*^
**Human development**		
Middle and Low		
High	6.29 (0.80-49.36)	0.08
Very High	5.30 (2.13-13.20)	<0.001^***^
**Health workforce**	0.98 (0.85-1.12)	0.74
**Year**	1.40 (1.20-1.65)	<0.001^***^

^^^p value <0.05^** ^p < 0.01 ^***^P < 0.001.

^^^^the cut-off point of timeliness gap is 3 days.

## Discussion

The reporting timeliness gap for avian flu and H1N1 outbreaks decreased significantly after 2003, during a time when newly emerging infectious diseases such as SARS appeared. Furthermore, the timeliness gap was significantly associated with civil liberties and human development indices but not health workforce density. Better civil liberties and human development were associated with longer delays in reporting of avian flu and H1N1 outbreaks.

Following the emergence of the global threat of SARS, countries became increasingly aware of the importance of timely reporting and the need for global collaboration in infectious disease control. The sharp decrease in the timeliness gap between ProMED-mail and the WHO report presented in 2003 and 2004, immediately following the SARS outbreak, is one reflection of this awareness. With the development of this strong consensus, the International Health Regulation 2005 was adopted. The fact that the average timeliness gap of H1N1 reports was shorter than the timeliness gap of avian flu reports demonstrates an increasing awareness of the seriousness of not disclosing information regarding a “public health emergency of international concern.” As a result, the yearly average reporting timeliness gap decreased significantly over time. However, this result does not reflect an improvement in the timeliness gap between the real time of an outbreak and the reporting time. In countries with inadequate surveillance systems or with insufficient civil liberties to release outbreak information, the shortened timeliness gap might be meaningless for public health concerns. Thus, further study is needed to understand the relationship between improvements in reducing the timeliness gap and the timely reporting of outbreaks.

Civil liberties were found to be negatively associated with reporting timeliness gap. It is undeniable that WHO reports are sometimes faster than ProMED-mail reports due to delays caused by countries’ insufficient civil liberties. However, given that ProMED-mail, the non-official global surveillance system, can freely receive outbreak information from the media at any time, it is understandable that the reporting timeliness gap between ProMED-mail and the WHO was larger in countries with better civil liberties. The finding that countries with greater civil liberties have longer timeliness gaps is consistent with our hypothesis that outbreak news is reported and disseminated more quickly and easily by the mass media in countries with greater civil liberties. However, official reporting can be very much delayed due to the multiplicity of stakeholders or the tardiness inherent in the reporting procedures of bureaucracies in official organisations and systems [29,30]. Thus, further study is needed to better understand the association between civil liberties and the reporting timeliness gap.

Similarly, human development was also found to be negatively associated with the timeliness gap. In general, countries with higher human development exhibited longer reporting timeliness gaps. Human development is defined by a combination of health, education and economic status indicators. According to previous studies, countries with greater civil liberties tend to be more democratic societies with better economic status, more widespread education and better overall health. Within a developed society, the complex process of administration undeniably slows the standard process and operation of official organisations and systems [29,30]. Therefore, countries with higher human development status have longer average reporting timeliness gaps. However, it is important to note that human development is not always a negative predictor of reporting timeliness. The shortest timeliness gap was found not in countries with low human development scores but rather in countries with medium human development scores. In other words, human development plays a positive role in improving reporting timeliness for countries in which development remained substandard. Relatively poor health technology or health resources in countries with low human development scores may account for this phenomenon. However, further research is needed to better understand the relationships between these factors.

No significant association between timeliness gap and health workforce was found in this study. Although a curved relationship between timeliness gap and health workforce was found, no significant association between timeliness gap and health workforce was indicated in our logistic regression analysis. The significant correlation between the health workforce density and the human development index might be one explanation for this finding. In addition, this phenomenon might reflect the importance of public health efforts other than health workforce density. Although an adequate health workforce is one of the fundamental components of any public health system, there are other important factors related to reporting timeliness, such as the quality of the health workforce, the awareness of unusual diseases among the health workforce, and the overall effectiveness of the healthcare system. Therefore, the lack of a significant association between timeliness gap and health workforce density in our study may be attributed to these additional factors.

One of the important limitations of this study was its cross sectional design. Although we analysed historical reporting timeliness gap data, we used human development and health workforce data from a single year to analyse the association between timeliness gap and the various factors. Thus, changes in human development status and health workforce density from 2003 to 2009 were not accounted for in this study. In addition, the sharp decrease in the timeliness gap between ProMED-mail and WHO reports in 2003 may produce spurious results. Moreover, the results of our study might overemphasise the effect of transparency on the reporting timeliness gap because we were not able to analyse other factors related to the timeliness of reporting, such as IT and communication infrastructure.

## Conclusions

This study found that countries were aware of the importance of the timely reporting of outbreak data, as represented by the adoption of IHR 2005, and that this awareness had a significant impact in improving the reporting timeliness gap. In addition, this study found significant associations between the reporting timeliness gap and civil liberties and human development. We have discussed this phenomenon previously, as well as the need to explore these issues in further studies. We suggest that efforts to increase transparency should be considered to further improve the global disease surveillance system. In the meantime, we must also consider that even with the best intentions, the release of disease information can be delayed by complex factors such as the number of stakeholders involved and the complex internal reporting systems of multiple official organisations.

## Abbreviations

SARS: Severe Acute Respiratory Syndrome; WHO: World Health Organisation; GOARN: Global Outbreak Alert & Response Network; IHR 2005: International Health Regulations; SYSRA: Systemic Rapid Assessment; HDI: Human Development Index; UNDP: United Nations Development Program; GDP: Gross Domestic Product.

## Competing interests

The authors declare that they have no competing interests.

## Authors’ contributions

FJT designed the study, collected the data, performed the statistical analysis, interpreted the data, and drafted the manuscript. ET participated in the data collection and in drafting the manuscript. CC Chan participated in formulating the study, interpreting the data and in drafting the manuscript. HT participated in interpreting the data and in drafting the manuscript. SM participated in interpreting the data and in drafting the manuscript. AR participated in interpreting the data and helped to revise the manuscript. All authors read and approved the final manuscript.

## Authors’ information

Feng-Jen Tsai holds a PhD in public health and an LLM degree in law. She is an assistant professor at Taipei Medical University (Program of Global Health and Development).

Eva Tseng received her MD and MPH from Tufts University. She is currently an internal medicine resident at Thomas Jefferson University Hospital in Philadelphia, PA.

Chang-Chuan Chan, ScD is a professor of the Institute of Occupational Medicine and Industrial Hygiene and the Chair of the Global Health Center, College of Public Health, National Taiwan University.

Hiko Tamashiro, PhD, DrPH, is a professor and the Chair of Department of Global Health and Epidemiology at the Hokkaido University Graduate School of Medicine.

Sandrine Motamed is an MD, MPH. She is a lecturer at the University of Geneva (Medical Faculty), chief resident at the University Hospital of Geneva and adjunct associate professor at the University of Hokkaido, Japan.

André C. Rougemont, MD, MPH is an honorary professor and the former Director of the Institute of Social and Preventive Medicine at the University of Geneva.
